# Allele-Specific Amplification in Cancer Revealed by SNP Array Analysis

**DOI:** 10.1371/journal.pcbi.0010065

**Published:** 2005-11-25

**Authors:** Thomas LaFramboise, Barbara A Weir, Xiaojun Zhao, Rameen Beroukhim, Cheng Li, David Harrington, William R Sellers, Matthew Meyerson

**Affiliations:** 1 Department of Medical Oncology, Dana-Farber Cancer Institute, Boston, Massachusetts, United States of America; 2 The Broad Institute of Harvard and MIT, Cambridge, Massachusetts, United States of America; 3 Departments of Biostatistics and Computational Biology, Dana-Farber Cancer Institute and Harvard School of Public Health, Boston, Massachusetts, United States of America; 4 Department of Medicine, Harvard Medical School, Boston, Massachusetts, United States of America; 5 Department of Pathology, Harvard Medical School, Boston, Massachusetts, United States of America; Millennium Pharmaceuticals, United States of America

## Abstract

Amplification, deletion, and loss of heterozygosity of genomic DNA are hallmarks of cancer. In recent years a variety of studies have emerged measuring total chromosomal copy number at increasingly high resolution. Similarly, loss-of-heterozygosity events have been finely mapped using high-throughput genotyping technologies. We have developed a probe-level allele-specific quantitation procedure that extracts both copy number and allelotype information from single nucleotide polymorphism (SNP) array data to arrive at allele-specific copy number across the genome. Our approach applies an expectation-maximization algorithm to a model derived from a novel classification of SNP array probes. This method is the first to our knowledge that is able to (a) determine the generalized genotype of aberrant samples at each SNP site (e.g., CCCCT at an amplified site), and (b) infer the copy number of each parental chromosome across the genome. With this method, we are able to determine not just where amplifications and deletions occur, but also the haplotype of the region being amplified or deleted. The merit of our model and general approach is demonstrated by very precise genotyping of normal samples, and our allele-specific copy number inferences are validated using PCR experiments. Applying our method to a collection of lung cancer samples, we are able to conclude that amplification is essentially monoallelic, as would be expected under the mechanisms currently believed responsible for gene amplification. This suggests that a specific parental chromosome may be targeted for amplification, whether because of germ line or somatic variation. An R software package containing the methods described in this paper is freely available at http://genome.dfci.harvard.edu/~tlaframb/PLASQ.

## Synopsis

Human cancer is driven by the acquisition of genomic alterations. These alterations include amplifications and deletions of portions of one or both chromosomes in the cell. The localization of such copy number changes is an important pursuit in cancer genomics research because amplifications frequently harbor cancer-causing oncogenes, while deleted regions often contain tumor-suppressor genes. In this paper the authors present an expectation-maximization-based procedure that, when applied to data from single nucleotide polymorphism arrays, estimates not only total copy number at high resolution across the genome, but also the contribution of each parental chromosome to copy number. Applying this approach to data from over 100 lung cancer samples the authors find that, in essentially all cases, amplification is monoallelic. That is, only one of the two parental chromosomes contributes to the copy number elevation in each amplified region. This phenomenon makes possible the identification of haplotypes, or patterns of single nucleotide polymorphism alleles, that may serve as markers for the tumor-inducing genetic variants being targeted.

## Introduction

Genomic alterations are believed to be the major underlying cause of cancer [1–3]. These alterations include various types of mutations, translocations, and copy number alterations. The last category involves chromosomal regions with either more than two copies (amplifications), one copy (heterozygous deletions), or zero copies (homozygous deletions) in the cell. Genes contained in amplified regions are natural candidates for cancer-causing oncogenes [4], while those in regions of deletion are potential tumor-suppressor genes [5]. Thus, the localization of these alterations in cell lines and tumor samples is a central aim of cancer research.

In recent years, a variety of array-based technologies have been developed to identify and classify genomic alterations [6–8]. Studies using these technologies typically analyze the raw data to produce estimates of total copy number across the genome [9–11]. However, these studies ignore the individual contributions to copy number from each chromosome. Thus, for example, if a region containing a heterozygous locus undergoes amplification, the question of which allele is being amplified generally remains unanswered. The amplified allele is of interest because it may have been selected for amplification because of its oncogenic effect. Data from array-based platforms have also been employed to identify loss-of-heterozygosity (LOH) events [12,13]. In these studies LOH is typically inferred to have occurred where there is an allelic imbalance in a tumor sample at the same site at which the matched normal sample is heterozygous. A complicating issue (particularly in cancer) is that the imbalance may be due to the amplification of one of the alleles rather than the deletion of the other, and thus LOH may not in fact be present.

Copy number analysis and LOH detection can both be improved by combining copy number measurement with allelotype data. In this paper, we present a probe-level allele-specific quantitation (PLASQ) procedure that infers allele-specific copy numbers (ASCNs) from 100K single nucleotide polymorphism (SNP) array [7] data. Our algorithm yields highly accurate genotypes at the over 100,000 SNP sites. We are also able to infer parent-specific copy numbers (PSCNs) across the genome, making use of the fact that PSCN is locally constant on each chromosome. (PSCNs here mean the copy numbers of each of the two parental chromosomes.) Our results also allow the distinction to be made between true LOH and (false) apparent LOH due to the amplification of a portion of only one of the chromosomes.

The PSCNs of 12 lung cancer samples that we initially analyzed reveal almost exclusively monoallelic amplification of genomic DNA, a result that we subsequently confirm in 89 other lung cell lines and tumors. Monoallelic amplification has previously been noted in the literature on the single gene level [14–16], wherein mutant forms of known oncogenes are amplified, while their wild-type counterparts are left unaltered. To our knowledge, this phenomenon has not previously been described on a genome-wide scale, though proposed mechanisms of amplification such as unequal sister chromatid exchange [17] would suggest monoallelic amplification as the expected result.

In addition, our ASCNs identify the SNP haplotypes being amplified. These haplotypes could conceivably serve as markers for deleterious germ line mutations via linkage disequilibrium. Indeed, the presence of monoallelic amplification makes such linkage studies statistically tractable (see Discussion).

## Results

### Model Specification and Justification

The 100K SNP array set [7] is a pair of arrays, corresponding to the HindIII and XbaI restriction enzymes, that together are able to interrogate over 100,000 human SNPs. Herein, we shall refer to the pair simply as the 100K SNP array. Its original intended use was to query normal human DNA at specific SNP sites, using a probe set of 40 25-mer oligonucleotide probes to interrogate each SNP. The aim is to identify which of the two alleles—arbitrarily labeled allele A and allele B—occurs in each chromosome at each SNP site. (Note that a diploid normal genome is implicitly assumed, though there are recent reports of copy number variation in normal cells [18,19].) An individual can therefore be genotyped at each SNP as either homozygous AA, homozygous BB, or heterozygous AB.

**Figure 1:**
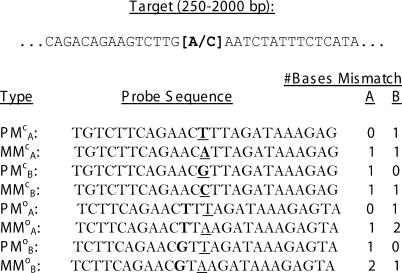
A Hypothetical Example of the Eight Probe Types in the 100K SNP Array [7] Each probe is a 25-mer designed to be at least partially complementary to a portion of the target fragment. In this diagram, the target contains an A (A allele)/C (B allele) SNP, as shown in brackets. The middle (13th) base of each probe is underlined, and the base corresponding to the SNP site is indicated in bold. The offset probes here are offset by two bases. From the sequences, one can count the number of bases that each probe mismatches each target allele (right columns).

The design of the array is such that each probe may be classified as either a perfect match (PM; perfectly complementary to one of the target alleles), or a mismatch (MM; identical to a perfect match probe except that the center base is altered so as to be perfectly complementary to neither allele). Further, probes may be subclassified according to whether they are complementary to allele A or allele B, yielding four types of probes: PM_A_, MM_A_, PM_B_, and MM_B_. A third subclassification is relevant. A probe may either be centered precisely at the SNP site, or may be offset by between one and four bases in either direction. This results in eight types of probes: 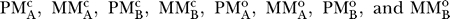 . Here the superscripts c and o denote “centered” and “offset,” respectively. Examples of each probe type and their base mismatch properties for a hypothetical SNP are shown in Figure 1. Our model relates a probe's intensity to the number of bases at which it mismatches each of the two allele targets (see below). Note that the eight probe types collapse to five types with respect to affinity for each allele, so that each of the 40 probes in a probe set may be classified as 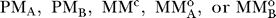 .

As a first step, we invariant-set normalized [20] all arrays to the same pair (one for the HindIII array and the other for the XbaI array) of baseline arrays using the dChip software (http://www.dchip.org). (Normalization is a standard first step in the analysis of microarray data, and is meant to eliminate unwanted artifacts such as differences in overall array brightness.) Our subsequent analyses are all based on a model that specifies probe intensity as a linear function of the copy numbers of both alleles. The underpinnings of this model are justified by empirical evidence that the signal from oligonucleotide probes is proportional to target quantity up until the point at which the probe becomes saturated [21].

A similar linear model has been well established for use with expression array data [22]. In our model, however, the proportionality parameters depend upon the numbers of bases at which the probe mismatches each target allele. Therefore, we specify the model for (normalized) probe intensity *Y_k_* of the *k*th probe in a fixed SNP's probe set as


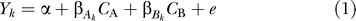


Here *C*_A_ and *C*_B_ are the copy numbers of the A and B alleles, respectively, in the sample being interrogated, and *A_k_* and *B_k_* denote the number of bases (either 0, 1, or 2) at which the *k*th probe is not perfectly complementary to the A and B targets, respectively. For example, it follows from Figure 1 that the model specifies a PM_A_ probe's intensity as α + β_0_ *C*_A_ + β_1_ *C*_B_ + *e*. The first term, α, represents background signal, which can arise from optical noise and nonspecific binding [23], and the error *e* is a normally distributed mean-zero term meant to capture additional sources of variation. Hence the model parameters are α, β_0_, β_1_, and β_2_. These parameters are allowed to be different for forward and reverse strands, and to vary from SNP to SNP, but are assumed to be constant within same-strand portions of probe sets and across different samples in a study. They effectively encode the binding affinities between the probes and targets for each SNP. Finally, our experience indicates that the two-base mismatch signal is essentially indistinguishable from background noise, and hence we set β_2_ = 0.

**Figure 2:**
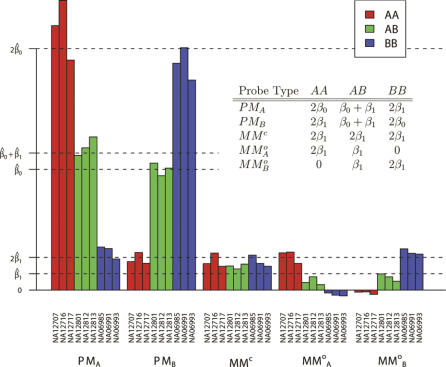
Average Intensities for Each Probe Type by Sample at a Single SNP (rs 2273762) The inset table gives the average background-subtracted intensities that would be predicted by our model. The actual background-subtracted mean intensity values (bar graph) in each sample closely agree with what is predicted (inset table).

From model equation 1 and Figure 1, it directly follows that the background-subtracted mean intensities in a normal sample should depend upon the genotype at the SNP in normal samples according to the inset table in Figure 2. We fit the model to data from nine samples—NA6985, NA6991, NA6993, NA12707, NA12716, NA12717, NA12801, NA12812, and NA12813—that were gathered as part of the International HapMap Project (http://www.hapmap.org). An example of the model fit is illustrated for a specific SNP (rs 2273762) in Figure 2. We estimated values α̂, β̂, and β̂_1_ for the parameters α, β_0_, and β_1_, along with genotyping calls for each sample using an expectation-maximization algorithm [24] (see Materials and Methods). In the figure, it can be seen that each probe classification's mean intensity agrees closely with that assumed by the model (inset table). This is an indication that the model provides a reasonably accurate description of the data.

### Genotyping of Normal Samples

We applied our method to the nine samples (see above) that were independently genotyped by centers in the International HapMap Project consortium. Nine different centers were involved in the genotyping of these samples. They employed a variety of platforms, including mass spectroscopy, enzymatic reactions, hybridization, and polymerase chain reaction (PCR)–based techniques. There are approximately 22,000 SNPs that are represented in both the 100K SNP array and the HapMap effort. In the nine samples we studied, a total of 1,198 SNPs were genotyped by two or more different HapMap centers, resulting in 10,782 sample SNP calls. The concordant calls among these multiply genotyped sample SNPs may be treated as being very close to a “gold standard” result, and we used these as a benchmark against which to evaluate the accuracy of our calls. Table 1 summarizes the comparison. The HapMap results have a 98.7% call rate. Among those called, the concordance rate between centers exceeds 99%. Our genotyping algorithm performs quite well, achieving a call rate of 99.27%, and disagreeing with the consensus HapMap genotyping for less than 1% of the calls. The results point to a very high rate of accuracy for our method, and speak well to the suitability of the model.

**Table 1:**
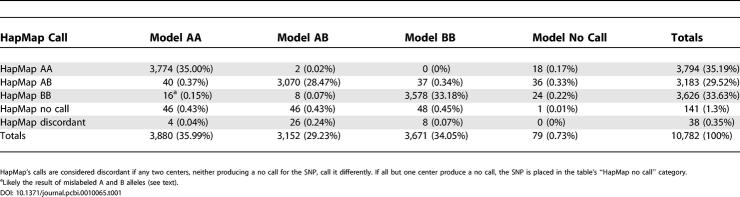
Concordance between Our Model's Calls and Those Made by More Than One Center in the International HapMap Project Effort

A feature of Table 1 that bears further comment is the fact that 16 sample SNPs were called AA by our algorithm and BB by the HapMap consortium. All 16 of these discrepancies occur in either of two SNPs, rs 1323113 or rs 2284867. Close inspection of the raw intensities of the 40 probes at each of these SNPs (data not shown) reveals a strong AA signal for the samples in question. A likely explanation is that the A and B labels were inadvertently switched for these two SNPs when Affymetrix matched its notation to the HapMap effort's alleles.

### ASCNs and PSCNs in Cancer DNA Samples

The distinction between ASCN and PSCN may be best understood by considering a hypothetical example of four consecutive SNPs in a genomic region with a total copy number of five. Suppose that the allele A copy numbers for the SNPs are four, zero, five, and one, respectively, leaving allele B copy numbers as one, five, zero, and four. These are what we mean by ASCNs. Taken individually, the ASCNs for the second and third SNPs are noninformative with regard to PSCN, as both the maternal and paternal chromosomes have the same alleles. However, the first and fourth SNPs both indicate that one of the parental chromosomes was amplified to a copy number of four, while the other is unaltered. Thus, we infer PSCNs of four and one for the entire genomic region containing the four SNPs. The ASCN at a SNP site may be viewed as a generalized genotype of the sample.

**Figure 3:**
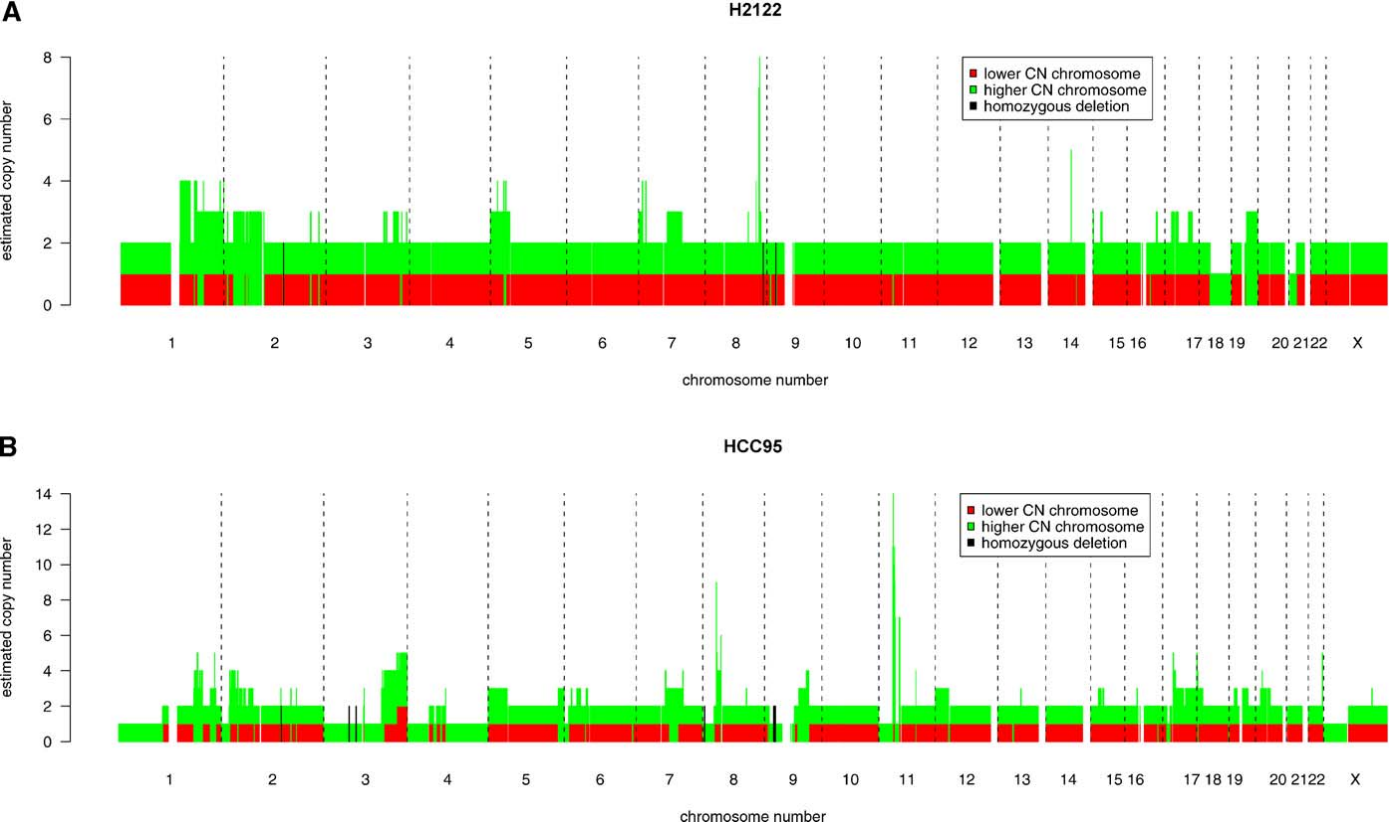
A Depiction of PSCN across the Genome for the Cell Lines H2122 and HCC95 In both graphs green indicates the higher copy number parental chromosome, and red indicates the lower copy number parental chromosome. The total height of each red/green bar indicates the total copy number at the corresponding SNP. Black bars represent homozygous deletions, where total copy number is zero.

We initially tested our PLASQ algorithm on a set of 12 lung cancer samples for which we have recently reported total copy number analysis [25], after calibrating the model on 12 normal samples. The cancer samples included one small cell primary tumor, two non-small cell primary tumors, and nine cell lines. Please refer to [25] and the Materials and Methods for additional details. All inferred homozygous deletions are provided in Table 2, while all inferred amplifications with total copy number of at least five are in Table 3. The genome-wide view of inferred PSCN is shown for the H2122 and HCC95 cell lines in Figure 3. The absence of minor chromosome copy numbers (red bars) at high levels on the plot shows that the amplifications are essentially monoallelic.

**Table 2:**
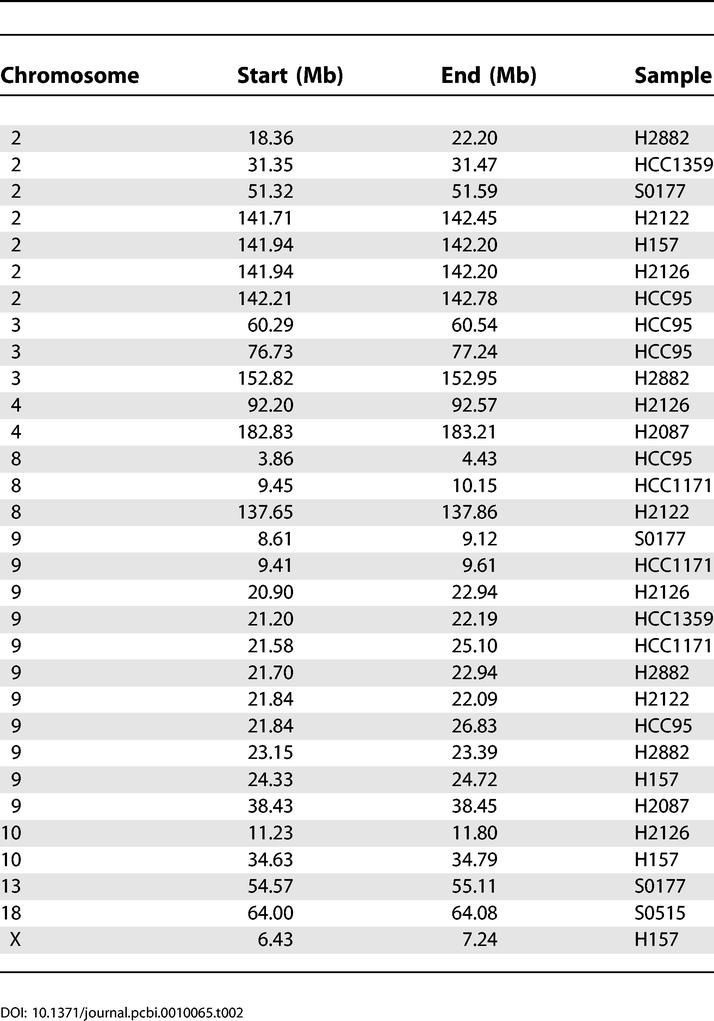
All PLASQ-Inferred Homozygous Deletions, across 12 Lung Cancer Samples

**Table 3:**
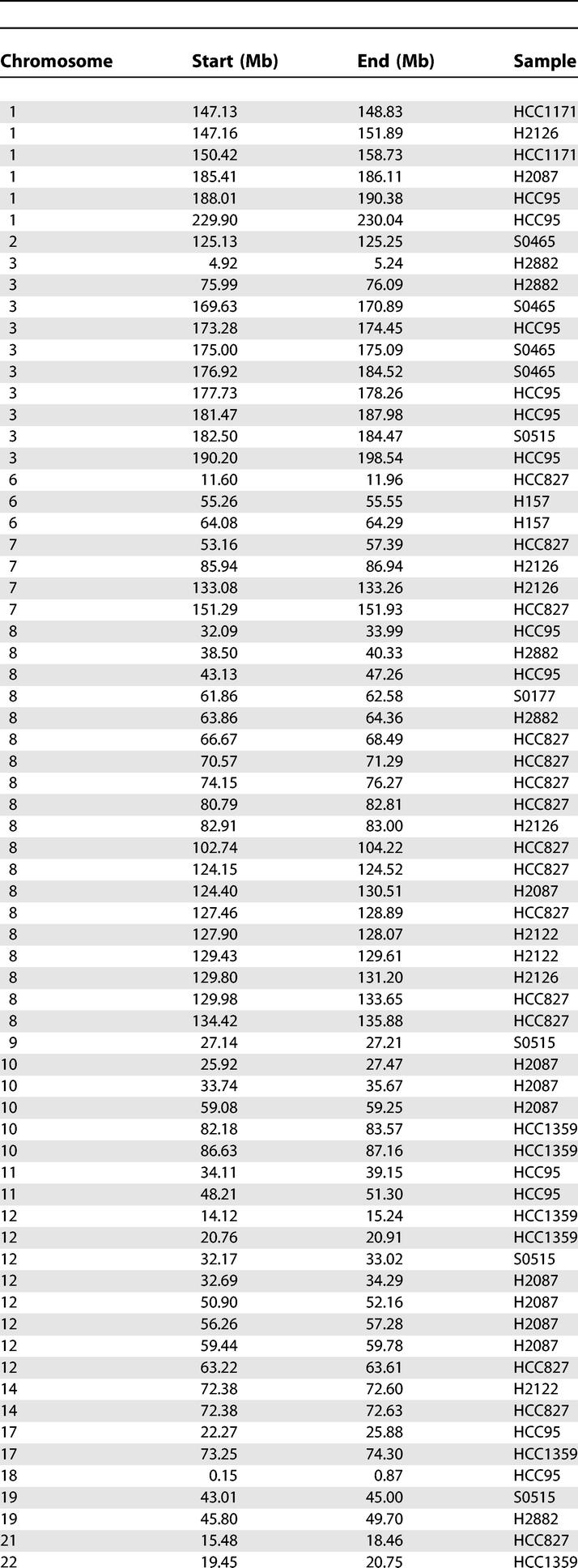
All PLASQ-Inferred Amplifications of Total Copy Number of at Least Five, across 12 Lung Cancer Samples

**Table 3:**
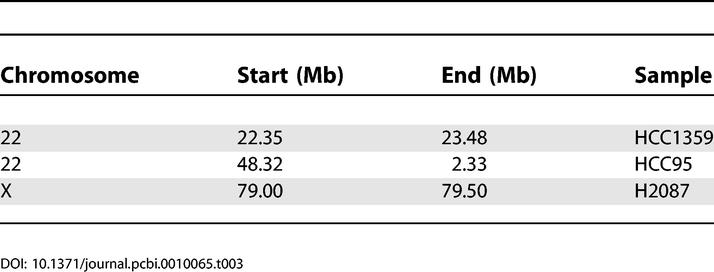
Continued

**Figure 4:**
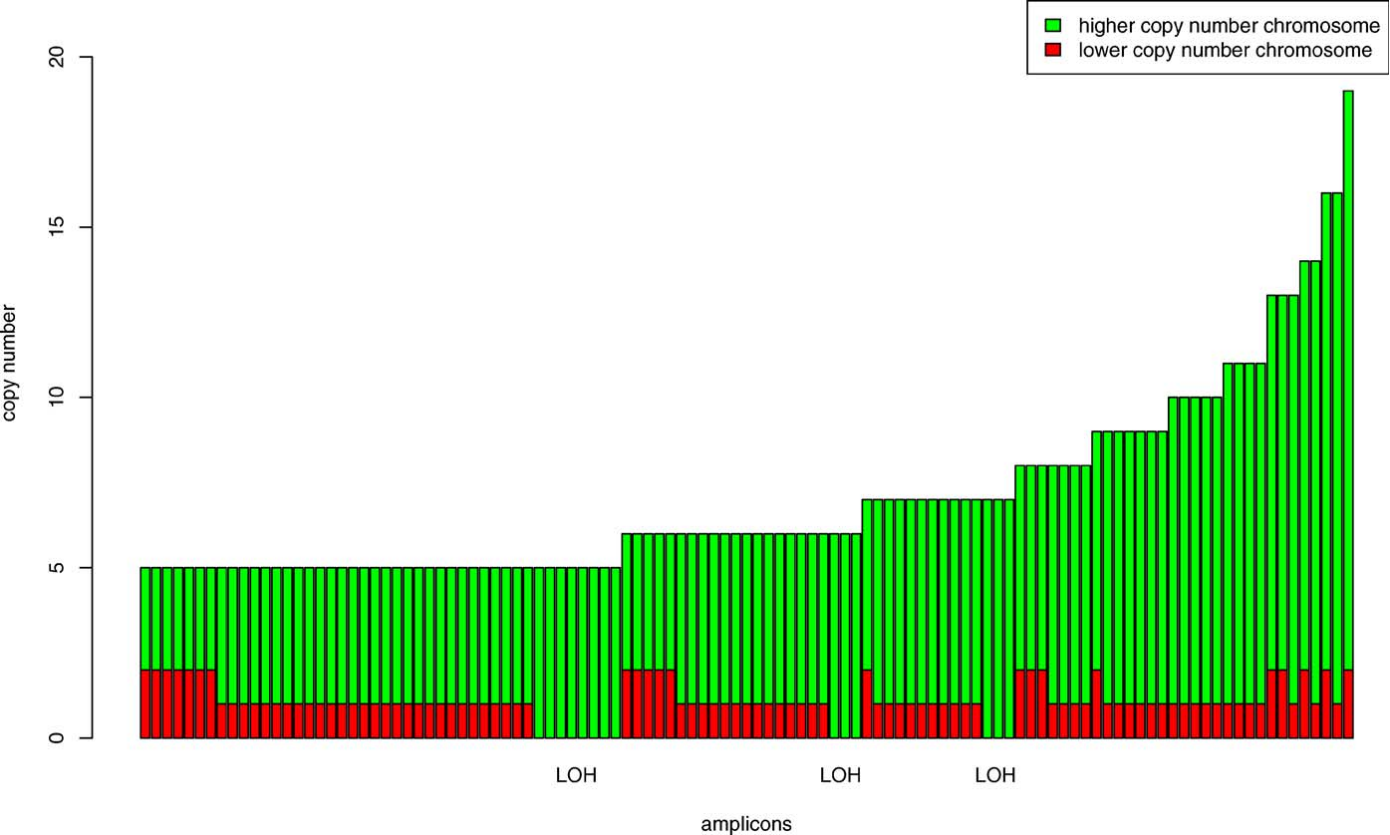
PSCNs for All Discovered Amplicons with PLASQ-Inferred Total Copy Numbers of at Least Five The height of each bar indicates the total copy number for that amplicon. The copy numbers for the parental chromosomes are represented by the red and green portions of each bar. LOH occurs, as indicated, where there is no red portion.

All amplicons with total inferred copy number of at least five, throughout all 12 samples, are shown in Figure 4. The most striking feature of this graph is the fact that the vast majority of amplifications exclusively involve only one of the two parental chromosomes. That is, amplification here is monoallelic. Also clear from the figure is the distinction between true LOH (bars with no red portion) and false LOH (bars partly red). We repeated our analysis on 89 other samples (data not shown), on which we similarly obtained the result that amplicons are almost entirely composed of only one of the two parental chromosomes.

To experimentally validate our PLASQ approach using an independent method, we applied allele-specific real-time PCR. ASCN analysis required changes to the standard copy number analysis by real-time PCR. Standard conditions using Taq polymerase caused the amplification of the target allele, as well as delayed amplification from the other SNP allele. The Stoffel fragment of Taq polymerase, which lacks that enzyme's normal 5′ to 3′ exonuclease activity, increases the specificity of the enzyme for the correct target [26,27]. This consequently increases the amplification delay enough to distinguish the two alleles and calculate accurate copy numbers.

In [25], we used standard real-time PCR to verify the total copy number for “recurrent” amplifications and deletions. We defined an event to be recurrent if it occurred in at least two samples, contained at least four SNPs, and was at least 5 kb in length. The comparison of our PLASQ analysis to both allele-specific and standard real-time PCR is given in Tables 4 and 5 for these recurrent events that occur in our initial 12 samples. PLASQ largely agrees with the PCR measurements for homozygous deletions (Table 4). For amplifications (Table 5), there is strong concordance between our estimates and the allele-specific PCR results. The rounded minor allele estimates differ by at most one copy in all but one case. With regard to major allele copy number inferences in Table 5, our estimates tend to be somewhat low, though they are always at elevated levels where the PCR results are. These discrepancies are likely the result of saturation effects that are well known in oligonucleotide arrays [28]. There is only one case where the total PCR estimate from [25] is lower than the PLASQ total. Here the allele-specific PCR results are in closer agreement with our inferred ASCN, indicating that this is an experimental error in the standard real-time PCR.

**Table 4:**
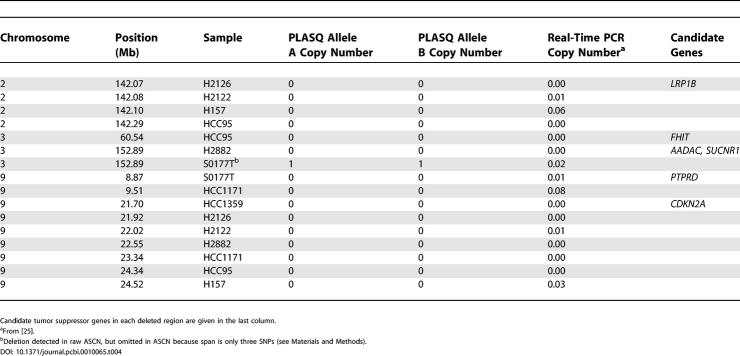
Comparison of Inferred ASCNs with PCR Results for Deletions

**Table 5:**
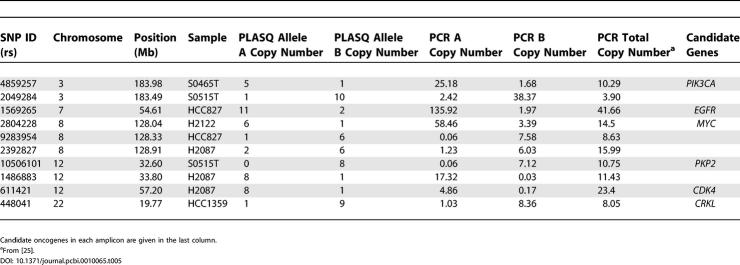
Comparison of Inferred ASCNs with PCR Results for Amplifications

One type of discrepancy in Table 5 stands out. In two cases, PLASQ infers an ASCN of one, whereas the experimentally determined copy number was essentially zero. One possible explanation is that our inference is correct and the low PCR estimates are attributable to experimental errors such as suboptimal primer sequences. On the other hand, our ASCN calls are somewhat vulnerable to the inherent noise in hybridization-based intensity measurements. At the single SNP level, deviations of one copy number in either direction may be difficult to detect because of this noise, resulting in slightly inaccurate ASCN calls. However, these inaccuracies are ameliorated in PSCN calls since we may “borrow strength” from neighboring SNPs' raw ASCNs because of the locally constant property of PSCN. Thus, for example, the LOH calls for regions will be very precise even when individual ASCN calls are slightly erroneous.

It is important to note that in all cases, the property of interest—the presence or absence of amplification or deletion in each chromosome—is clearly detectable with our method, as all approaches agree in this regard. Finally, in order to assess the accuracy of our determination of amplicon and deletion boundaries, we compared the results that were determined in [25] using an algorithm implemented in the dChipSNP computational platform [9] to our results. The comparison is shown in Table 6 for the events in Tables 4 and 5. In most cases, our estimated alteration boundaries correspond exactly to those inferred by dChipSNP. Events for which the two approaches differ in their inferences could be due to procedural differences such as varying copy number thresholds used to determine whether or not a gain should be called an amplification.

**Table 6:**
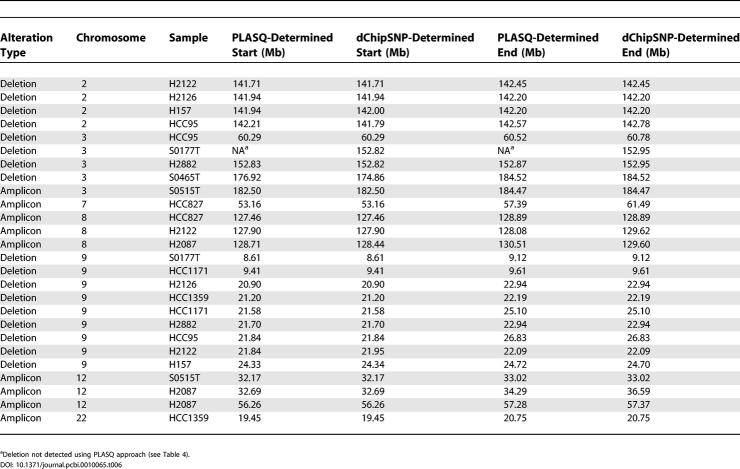
Comparison of PLASQ-Inferred Lesion Boundaries with Those from [25]

### Amplification of *EGFR* Mutant

In order to determine whether amplification could target, in a monoallelic fashion, an activating mutation in one of our samples, we examined sequence data for the *EGFR* gene. It was shown in [25] that the HCC827 cell line harbors the E746 A750del deletion mutant. This is a known activating mutation [29,30], and our result in Table 5 predicts ASCNs of 11 and two at this locus. It was interesting, therefore, to determine whether the greatly amplified chromosome is the one harboring the mutation. To answer this question, we performed quantitative PCR experiments that are able to differentiate the wild-type copies from the mutant copies (see Materials and Methods). The wild-type allele was found to be unamplified (PCR estimate 0.80), while the total PCR copy number was 39.78. Thus, our method uncovered a targeted amplification of an activating mutant allele over its wild-type counterpart.

## Discussion

Many genomic events of interest are easily placed in the context of ASCN and PSCN. LOH at a SNP site occurs where one of the PSCNs is zero. Monoallelic amplification occurs at loci where one parental chromosome has a copy number less than two and the other has a copy number greater than one. We have demonstrated that these events, among others, may be identified though ASCN and PSCN from 100K SNP array data. Examining array data from over 100 lung cancer samples, we have found that amplifications are overwhelmingly monoallelic. Current understanding of the mechanisms behind amplification in tumorigenesis would suggest this as an expected result. For example, Herrick et al. [17] describe mechanisms that would all lead to monoallelic amplification in genes. To our knowledge, however, this phenomenon has not been demonstrated on a genome-wide scale in the literature.

Previous studies have demonstrated monoallelic amplification at specific genes. Hosokawa and Arnold [14] found two tumor cell lines in which a mutant allele of *cyclin D1* is amplified but the wild-type copy is not. Zhuang et al. [16] uncovered a similar trend in 16 renal carcinoma tumors heterozygous for a *MET* mutation, and a study of 26 mouse skin tumors found 16 with a mutant *HRAS* homolog allele amplified but none with the wild-type allele amplified [15]. Using our procedure, we have uncovered (and validated) an *EGFR* example in one of our samples. These cases highlight the targeting of one genetic variant for amplification over another at a heterozygous site, presumably in order to give the cell growth advantage. However, further studies involving a larger set of tumors are necessary to uncover multiple instances of the transforming variant being the amplification target. A large number of such cases would provide compelling evidence for the biological significance of allele-specific amplification of genes. In some studies these monoallelic amplifications may be erroneously called LOH because of the allelic imbalance. Our approach was able to determine that, in most cases, the minor allele is not in fact deleted, and thus LOH has not occurred.

ASCN information may be used to identify SNP haplotypes in cancer cell amplicons. This haplotype structure determination has important applications for uncovering candidate oncogenes and tumor suppressor genes. The applications may be understood in the context of a recent study [31] that characterizes the genome as consisting of haplotype blocks—regions with few distinct haplotypes commonly observed in human populations—separated by recombination “hotspots.” Indeed, consider an inherited variant that predisposes a cell toward tumor growth and is selected for amplification. Many SNP sites located in the same haplotype block would be amplified along with the variant. One may determine the haplotype of the amplicon via ASCN. The SNP haplotype in the same block as the gene, therefore, may serve as a marker for the variant through genetic association studies [32]. We point out that, were it not for monoallelic amplification, this endeavor would be far more difficult, for if both parental chromosomes were amplified then both haplotypes would be candidate markers for the deleterious variant. Statistically, the power to detect association would be significantly compromised.

Our method produces, in addition, highly accurate genotype calls in normal cells. Analyzing sample SNPs that were genotyped by at least two independent groups, we had over 99% agreement with their concordant calls. Given the strength of our results, we are now working to apply the model to data from oligonucleotide resequencing arrays [33].

Note that our procedure is does not take into account all types of genomic alterations. For example, it would be somewhat confounded by a translocation event. A translocation would induce a loss of the “local constancy” property of total copy number. Similarly, point mutations are not detectable with our approach, and in fact could adversely affect copy number measurements if they were to occur near 100K SNP sites. Still, we feel that these limitations do not severely impact the applicability of the method.

The structure of our model suggests a very useful extension. A common problem in analyzing the genomic content of tumor cells is that of stromal contamination—the presence of normal cells in the sample. Stromal contamination makes accurate copy number determination difficult because the quantity measured is actually a weighted average of the normal and cancer cell copy numbers. Mathematically, the sample's ASCNs at a fixed SNP site may be expressed as


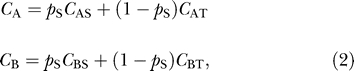


where *p*_S_ is the (unknown) proportion of stroma, *C*_AS_ and *C*_BS_ are the ASCNs of the stromal cells, and *C*_AT_ and *C*_BT_ are the (unknown) ASCNs in the tumor. We may treat *C*_AS_ and *C*_BS_ as known, since a matched normal sample may be genotyped at the SNP. Thus, replacing *C*_A_ and *C*_B_ in our model with the expressions in equation 2 above gives each probe's intensity as a function of true cancer cell ASCNs and proportion of stromal content. Although beyond the scope of this paper, this is an intriguing bioinformatic approach to a pervasive experimental problem.

In summary, we have presented a procedure, termed PLASQ, that is not only able to localize copy number alterations in cancer cells, but can also identify each chromosome's contribution to these alterations as well as the SNP haplotypes in each event. Our approach has been validated using a variety of independent experimental techniques. We have also described several applications and extensions of our methods, and we have demonstrated that chromosomal amplifications in human lung cancer are monoallelic. Finally, it has come to our attention that, while this work was under review, a pair of papers [34,35] describing methods to infer PSCN from 100K SNP array data was published. The approaches differ from ours, and appear to require matched normal samples.

An R [36] package, downloadable at http://genome.dfci.harvard.edu/~tlaframb/PLASQ, contains procedures and data described in this work.

## Materials and Methods

**Figure 5:**
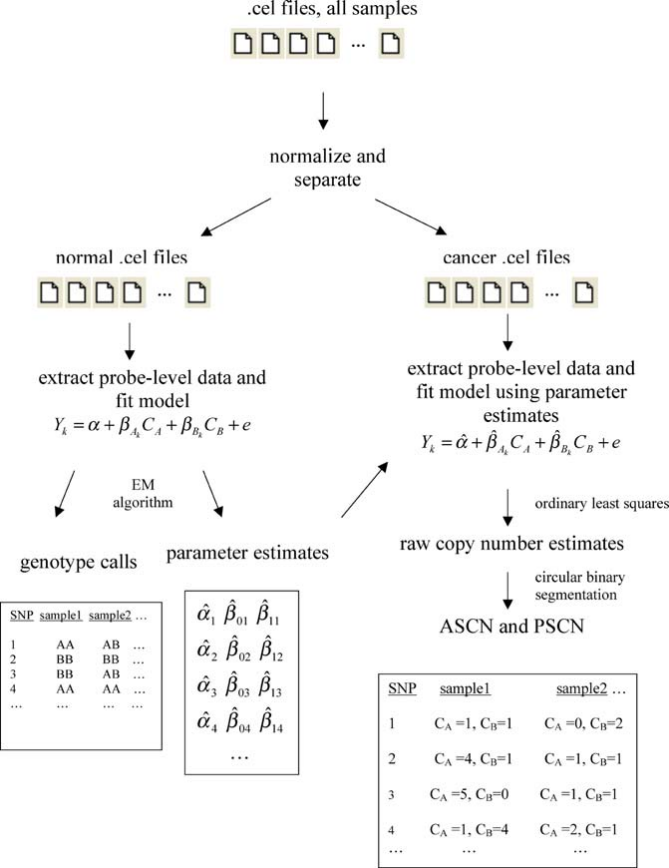
The PLASQ Procedure for Determining ASCN and PSCN from the .cel Files After normalizing signal intensities from all samples, the model is first fit to the normal samples' data to produce both genotype calls and parameter estimates at each SNP site. The latter are used in the model as applied to the data from the cancer samples. Ordinary least squares fitting produces raw ASCN estimates at each SNP. The corresponding raw total copy number estimates are smoothed using circular binary segmentation. Finally, further processing yields our final ASCN and PSCN inferences (see Materials and Methods). EM algorithm, expectation-maximization algorithm.

The PLASQ procedure for genotyping normal and aberrant samples (thereby obtaining ASCN and PSCN), beginning with the SNP array .cel files, is outlined in Figure 5. Details of each step are given below and in the Results.

### DNA samples.

We obtained the Affymetrix .cel files from all lung cancer tumors and cell lines analyzed in [25]. In our analysis, we used the same raw probe-level data that were generated from the experiments in that study. For initial analysis, we selected the cell lines H157, H2087, H2122, H2126, H2882, HCC95, HCC827, HCC1359, and HCC1171, as well as tumors S0177T, S0465T, and S0515T. These 12 samples were chosen because each was found [25] to harbor at least two of the copy number alterations that were considered recurrent. We subsequently applied our approach to the remaining 89 tumors and cell lines in that study. Additionally, the 12 normal samples from that paper were employed in the study. Details about the preparation, hybridization, and image acquisition for all samples may be found in [25], and all .cel files are available at http://research2.dfci.harvard.edu/dfci/snp/. We obtained the HapMap samples' .cel files from the Affymetrix Web site (http://www.affymetrix.com).

### Normal sample genotyping.

In this case, for each sample the value of *C*_A_ at a SNP is either zero, one, or two. The value of *C*_B_ is completely determined by *C*_A_, as *C*_A_ + *C*_B_ = 2. Thus, we may think of each sample SNP as being in one of three states, corresponding to the AA, AB, and BB genotypes. These states are not known a priori, and neither are the values of α, β_0_, and β_1_. We employ an expectation-maximization algorithm [24] at each SNP to infer the genotypes and estimate the parameters. Briefly, we first initialize the probabilities of the three genotypes of each sample using a crude *t*-test approach. Based on these initial “guesses,” we apply ordinary least squares [37] to our model, finding the maximum likelihood estimates of the parameters α, β_0_, and β_1_ (the M step). Next, based upon these estimates, we re-infer the genotype probabilities of each sample using the expected values of the indicator variables for each of the three possible genotypes (the E step). These two steps—maximization and expectation—are iterated until the approximated values of all unknowns converge. The result of this procedure is an estimated probability of each genotype along with parameter estimates. The algorithm's call at each sample SNP is the genotype with the maximum final estimated probability, unless the maximum falls under a user-defined threshold (the default is 99%), in which case a “No Call” is given. We subsequently use the final parameter estimates α̂, β̂, and β̂_1_ of α, β_0_, and β_1_, respectively, in the application of the model to data from cancer cells (see below).

### Total copy number in cancer DNA samples.

In an aberrant sample, copy numbers of the A and B alleles are no longer constrained to sum to two at each SNP. After calibrating the model on normal samples as described above, we replace the parameters α, β_0_, and β_1_ in our model with their estimates at each SNP. We directly apply least squares estimation to find raw inferences (“raw” because we do not yet exploit local constancy of total copy number) of the A and B copy numbers at each SNP. These rough measures are referred to as the raw ASCNs. While the ASCNs are not locally constant in a sample, their pairwise sums *C*_A_ + *C*_B_ are. We therefore input the pairwise sums of the raw ASCNs at each SNP into the circular binary segmentation algorithm [38] to infer total copy number. This smoothing algorithm exploits the fact that chromosomal alterations typically occur in segments containing several SNPs. Briefly, circular binary segmentation searches for locally constant sections by recursively splitting chromosomes into candidate subsegments and computing a maximum *t*-statistic that reflects differences in mean total raw copy number between subsegments. The reference distribution for this statistic, estimated by permutation, is used to decide whether or not to permanently split at each stage. The result is a segmentation of each chromosome in a sample, where the total copy number is deemed constant within each segment. Our raw total copy number of a segment is the mean of the pairwise sums of the raw ASCNs of all SNPs in the segment.

### PSCNs and ASCNs.

The circular binary segmentation algorithm divides each sample's genome into segments, each assumed to have the same total copy number. Consider a segment with *n* SNPs and a raw total copy number *T*_raw_. We infer PSCN for the segment as follows. If *n* < 4, we consider *T*_raw_ to be too noisy due to the small number of observations, and infer PSCNs (1, 1). For *n ≥* 4, if *T*_raw_ ≤ 0.35, the segment is called a homozygous deletion, giving PSCNs (minor chromosome, major chromosome) = (0, 0). If 0.35 < *T*_raw_ ≤ 1.35, we call a heterozygous deletion with PSCNs (0, 1). If *T*_raw_ > 1.35, our inferred total copy number *T* is simply *T*_raw_ rounded to the nearest integer (or to two if 1.35 < *T*_raw_ ≤ 2.5), and we proceed as follows.

Let *A*_1_, *A*_2_,…, *A_n_* and *B*_1_, *B*_2_,…, *B_n_* denote the raw ASCNs for the *n* SNPs in a segment. We consider a SNP *i* to be homozygous if minimum (*A_i_, B_i_*) ≤ 0.5. We must first consider the possibility that one of the parental chromosomes is deleted while the other is amplified, i.e., the SNP may be homozygous either because it was homozygous in the normal cell, or because of LOH. Since the average heterozygosity rate for SNPs on the array is 0.3 [39], the probability of a randomly chosen SNP being homozygous is 0.7. Thus, we model the number of homozygous SNPs in a segment without chromosomal deletion as a binomial (*n,* 0.7) random variable *X*. The resulting hypothesis test would reject the null hypothesis of no LOH at the α level if


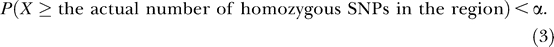


Making a conservative Bonferroni correction for multiple testing on the total number of segments *s,* we assume deletion of one chromosome if the null hypothesis is rejected at the α = 0.05/*s* level. In this case, our inferred PSCNs are (0, *T*). Otherwise, note that (as discussed in Results) homozygous SNP sites are noninformative with regard to PSCN. Thus, we temporarily ignore those SNPs, leaving *m* SNPs *(m ≤ n)* whose raw ASCNs we relabel *A*_1_, *A*_2_,…, *A_m_* and *B*_1_, *B*_2_,…, *B_m_*. Our inferred minor chromosome PSCN is then


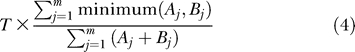


rounded to the nearest integer. In order to ensure that total copy number is *T,* the inferred major chromosome PSCN is *T* − (inferred minor chromosome PSCN).

Once PSCNs are determined, the ASCNs follow immediately from these and the raw ASCNs. The homozygous SNPs (determined as in the paragraph above) are assigned the allele with the larger raw ASCN. Heterozygous SNPs are assigned ASCNs so that the allele with the larger raw ASCN has the copy number of the major parental chromosome.

### PCR-based copy number validation.

Relative copy numbers for both alleles of a SNP site were determined by quantitative real-time PCR using both a PRISM 7500 Sequence Detection System (96 well) and a PRISM 7900HT Sequence Detection System (384 well) (Applied Biosystems, Foster City, California, United States). Real-time PCR was performed in 25-μl (96 well) or 12.5-μl (384 well) reactions with 2 ng or 1 ng, respectively, of template DNA. SYBR Green I (Molecular Probes; Eugene, Oregon, United States) and the Stoffel fragment of Taq polymerase (Applied Biosystems) [27] were used for the PCR reaction. The reaction mix used was as described previously [27], with the following exceptions: 3U of Stoffel polymerase, 100 μM dUTP, and 0.5 μM ROX (Invitrogen, Carlsbad, California, United States) were used per reaction. Primers were designed with the help of Primer 3 (http://frodo.wi.mit.edu/cgi-bin/primer3/primer3_www.cgi) and synthesized by Invitrogen. For each SNP site three primers were designed, one common for the region and two designed with the 3′ base of the primer specific for each SNP allele. The common primer plus one of the SNP-specific primers were used for each PCR reaction (0.3 μM each). Primer sequences are available upon request. PCR conditions were as follows: 2 min at 50 °C, 15 min at 95 °C, followed by 47 three-step cycles of (20 s at 95 °C, 20 s at 60 °C, and 30 s at 72 °C). The standard curve method was used to calculate the copy number of each allele of a target SNP site in the tumor DNA sample relative to a reference, the Line-1 repetitive element whose copy number is similar between both normal and cancerous cells. Quantification was based on standard curves from a serial dilution of human normal genomic DNA. The relative target copy number level for each allele of a SNP target site was normalized to normal human genomic DNA, heterozygous for that particular SNP site, as calibrator. Changes in the target allele copy number relative to the Line-1 and the calibrator were determined using the formula (*T*_target_/*T*_Line-1_)/(*C*_target_/*C*_Line-1_), where *T*_target_ and *T*_Line-1_ are the DNA quantities from tumor by using the target allele and Line-1, and *C*_target_ and *C*_Line-1_ are the DNA quantities from the calibrator by using the target allele and Line-1. The copy number of both alleles for each SNP site was determined in this way.

Real-time PCR was also used to determine the relative copy number of the two *EGFR* alleles in the HCC827 cell line, which contains the E746 A750del mutation and an amplification of the *EGFR* region. Real-time PCR was performed with the Stoffel fragment of Taq polymerase using reaction mix and conditions described above. The standard curve method was used to calculate the total copy number of the *EGFR* gene and the copy number of the wild-type allele in the HCC827 DNA sample normalized to Line-1 and a normal reference DNA. The primer pairs consisted of one common reverse primer, with one forward primer that would bind both *EGFR* alleles (wild-type and mutated) and one forward primer specific for the wild-type allele. The primer specific for the wild-type *EGFR* allele was designed so that the 3′ end was located within the DNA deleted by the E746 A750del mutation. Two PCR reactions were performed: one that gave total *EGFR* copy number (using primer that binds both alleles) and one that gave only wild-type *EGFR* copy number (using primer specific for wild-type *EGFR*).

## Supporting Information

### Accession Numbers

The NCBI Entrez Gene (http://www.ncbi.nlm.nih.gov/entrez/query.fcgi?db=gene) accession numbers for the genes discussed in this paper are *cyclin D1* (595), *EGFR* (1956), *HRAS* (3265), and *MET* (4233).

## Abbreviations

ASCN: allele-specific copy number
LOH: loss of heterozygosity
MM: mismatch
PCR: polymerase chain reaction
PLASQ: probe-level allele-specific quantitation
PM: perfect match
PSCN: parent-specific copy number
SNP: single nucleotide polymorphism
